# Efficacy of Etidronic Acid for Smear Layer Removal: A Systematic Review of In Vitro Studies

**DOI:** 10.3390/jfb17010048

**Published:** 2026-01-16

**Authors:** María-Inmaculada Vidal-Montolío, José Luis Sanz, James Ghilotti, Sofía Folguera, Carmen Llena

**Affiliations:** Department of Stomatology, Faculty of Medicine and Dentistry, Universitat de València, 46010 Valencia, Spain; vimain@alumni.uv.es (M.-I.V.-M.); james.ghilotti@uv.es (J.G.); sofia.folguera@uv.es (S.F.); llena@uv.es (C.L.)

**Keywords:** etidronic acid, irrigation, continuous chelation, sodium hypochlorite, ethylenediaminetetraacetic acid, in vitro, systematic review

## Abstract

Irrigation plays a crucial role in the success of root canal treatment; however, currently, no standardized irrigation protocols exist, particularly regarding the optimal sequence for smear layer removal. This systematic review aimed to determine which irrigation protocol achieves superior smear layer removal: traditional sequential irrigation with sodium hypochlorite (NaOCl) followed by ethylenediaminetetraacetic acid (EDTA), or irrigation with etidronic acid, either combined with NaOCl in continuous chelation or used as a final irrigant. Continuous chelation with etidronic acid may be clinically advantageous in daily practice, as it would facilitate workflow by using a single irrigating solution without compromising the efficacy of the irrigation process. A comprehensive electronic search was conducted in Medline, Embase, Cochrane, Scopus, and Web of Science, last updated in August 2025. In vitro studies were selected according to predefined PICO-based criteria. Two reviewers independently screened the studies and extracted data, with an inter-rater agreement of 0.92 using the Kappa index. Risk of bias was evaluated using a modified CONSORT checklist for in vitro studies on dental materials. The average item compliance of the included studies was 58%. The maximum score was 73% and the minimum was 47%. Twenty studies met the inclusion criteria. Etidronic acid used in continuous chelation showed equal or superior smear layer removal compared with sequential irrigation in nine of ten studies. Conversely, when used as a final irrigant, etidronic acid demonstrated inferior performance in more than half of the studies, particularly in the apical third. Based on the available evidence, etidronic acid in continuous chelation appears as effective as, or more effective than, traditional NaOCl–EDTA sequential irrigation.

## 1. Introduction

The primary objective of root canal treatment is to achieve healing of apical periodontitis or to prevent its development [1]. The success of this therapy largely depends on effective disinfection of the root canal system [2]. However, this process may be compromised by the presence of the smear layer, a three-dimensional layer formed on radicular dentin walls during root canal shaping [3] consisting of both organic and inorganic components [4].

The removal of the smear layer is essential [5], as its persistence reduces the penetration of irrigants and intracanal medicaments into dentinal tubules [6,7], it may act as a microbial reservoir promoting endodontic failure [8], and its removal enhances the sealing ability of root canal filling materials [9].

Since no single irrigant can effectively act on both the organic and inorganic components of the smear layer, at least two solutions must be employed [10]. Although no clear standardized protocols are available, the most widely used approach is sequential irrigation with sodium hypochlorite (NaOCl, 0.5–6%) and ethylenediaminetetraacetic acid (EDTA, 17%) [11,12,13,14]. This combination has demonstrated high efficacy and favorable long-term clinical outcomes [15]. Nevertheless, it is not without drawbacks: EDTA reacts with NaOCl, reducing its antimicrobial activity and tissue-dissolving capacity [16]. Moreover, EDTA has been associated with marked erosion of radicular dentin [17,18,19,20]. Such alterations are undesirable, as changes in dentin strength and mechanical properties may compromise long-term tooth survival [21]. Additionally, the use of multiple solutions increases time and material consumption and complicates workflow [22]. To avoid these drawbacks, a new irrigation concept known as continuous chelation has been introduced [23]. It consists of using a single solution throughout the entire root canal treatment: a combination of NaOCl and etidronic acid, a mild and compatible chelating agent.

Etidronic acid, also known as etidronate or 1-hydroxyethane-1,1-diphosphonic acid (HEDP), is a bisphosphonate used in medicine as a systemic medication for conditions such as osteoporosis, Paget’s disease, or neoplastic diseases with osteolytic bone destruction [24,25]. There are currently commercial solutions on the market for use as irrigants in endodontics, such as DualRinse (Medcem, Weinfelden, Switzerland), Twin Kleen (MaarcDental, Maharashtra, India), and Chloroquick (Neelkanth Dental and Surgical Factory, Jodhpur, India).

The presence of the chelating agent throughout the process could prevent the appearance of the smear layer or eliminate it immediately after it appears [26]. This would prevent the inactivation of part of the available chlorine that occurs when NaOCl comes into contact with the organic component of dentinal debris and would facilitate its penetration into the dentinal tubules [27]. As an added benefit, using a single solution throughout the entire root canal treatment would reduce the time and material required and simplify workflow.

The use of etidronic acid as a final irrigant, replacing EDTA, has also been investigated in the literature [28,29]. Current evidence indicates that etidronic acid in combination with NaOCl does not affect the antibacterial capacity of the latter [30,31,32,33,34]. Literature has also demonstrated a lower demineralizing effect on root dentin than EDTA [35]. However, the chelating action of etidronic acid is relatively weak [36]; therefore, there are doubts about its effectiveness in removing the smear layer compared to a strong chelating agent such as EDTA.

Accordingly, the objective of this systematic review is to compare traditional sequential irrigation (NaOCl and EDTA) with etidronic acid irrigation (combined with NaOCl in continuous chelation or as a final irrigant) in terms of their ability to remove the smear layer from inside the root canal.

The working hypothesis tested in this systematic review is that irrigation with etidronic acid, particularly when used in continuous chelation, differs in its ability to remove the smear layer compared with traditional sequential irrigation with sodium hypochlorite and EDTA. Conversely, the null hypothesis is that there are no differences in smear layer removal between traditional sequential irrigation with sodium hypochlorite and EDTA and irrigation with etidronic acid, either used in continuous chelation or as a final irrigant.

## 2. Materials and Methods

This systematic review followed the guidelines of the PRISMA 2020 statement (Preferred Reporting Items for Systematic Reviews and Meta-analyses) [37]. The protocol was previously registered on the platform Open Science Framework (OSF) (https://doi.org/10.17605/OSF.IO/9SCUK).

### 2.1. Search Strategy

A comprehensive electronic search was conducted in the following databases: Medline (via PubMed), EMBASE, Cochrane, Scopus, and Web of Science on 26 November 2024, with an update on 10 August 2025. No language or year restrictions were applied to the search. In addition, the reference lists of eligible studies were manually reviewed to identify additional articles.

The search strategy was designed based on previous studies in the area and their most cited descriptors. Consequently, the following terms were combined for each database: ‘root canal treatment,’ ‘endodontics,’ ‘in vitro,’ ‘etidronic acid,’ ‘HEDP,’ ‘HEBP,’ ‘chelation therapy,’ ‘chelating agents,’ ‘root canal irrigants,’ and ‘smear layer.’ Both MeSH terms and uncontrolled descriptors were used, combined using the Boolean operators ‘AND’ and ‘OR’ to refine the search. The electronic search strategy and its results are illustrated in Supplementary Table S1.

### 2.2. Eligibility Criteria

The eligibility criteria were designed following the PICO (S) strategy, as follows: Population (P): extracted human teeth; Intervention (I): irrigation with etidronic acid; Comparison (C): irrigation with NaOCl and EDTA; Outcome (O): removal of the smear layer assessed by scanning electron microscopy; Study design (S): in vitro studies.

### 2.3. Study Selection

The resulting records from the study search process were imported to Mendeley 1.19.8 reference management software (Elsevier, Amsterdam, Netherlands), where duplicate records were manually discarded. Then, two researchers (M.-I.V.-M. and C.L.) independently reviewed the titles and abstracts to apply the eligibility criteria. Discrepancies were resolved by consensus. The agreement between researchers was calculated using the Kappa index, which yielded a result of 0.92. Lastly, the full text of the selected studies was accessed to confirm their compliance with the eligibility criteria.

### 2.4. Quality Assessment

The risk of bias was determined using the CONSORT guideline adapted for in vitro studies on dental materials [38]. The criteria evaluated included, among others: providing an appropriate context and justifying the need for the study, defining specific objectives and/or hypotheses, describing the intervention in sufficient detail to allow for its replication, fully defining all variables studied, calculating the sample size, describing the method used to randomise the samples, describing the statistical calculation used, addressing the limitations of the study and potential sources of bias, and mentioning the source of funding.

When a criterion was met, it was rated as ‘Yes’ and, if not, as ‘No’. The percentage of compliance for each study with the quality scale was then calculated as follows: (number of items met/total number of items) × 100. The percentage of compliance for each of the items was also calculated: (number of articles meeting the item/total number of studies) × 100.

### 2.5. Data Extraction

The relevant data from the included studies were compiled and categorized according to the following variables: author/year, sample characteristics (total sample size and by groups and selected dental group), sample preparation (decoronation or not of the samples, total root length and method for closing the apical foramen), instrumentation technique (method for determining working length, instrumentation system, final apical caliber), irrigants compared and their concentration, irrigation technique (method, guidelines during instrumentation—volume and working time, final irrigation guidelines—volume and working time, and irrigant activation technique), evaluation of results for the study of smear layer (evaluation scale, root thirds in which the samples were photographed, magnification at which they were evaluated under the scanning electron microscope) and results of all variables studied (mean and *p*-value).

## 3. Results

### 3.1. Database Search and Study Selection

**Figure 1:**
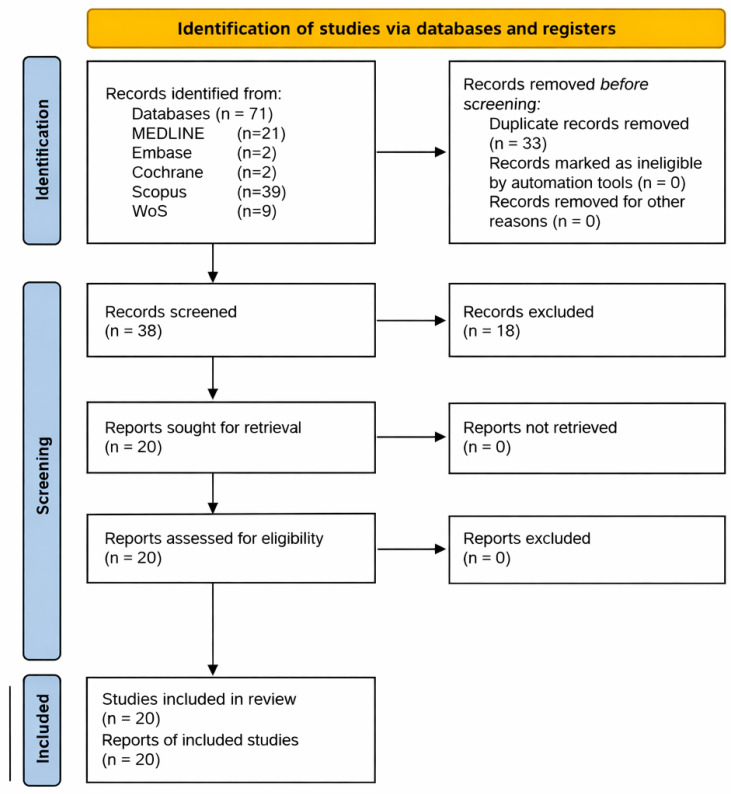
PRISMA 2020 flow diagram representing the study selection process.

The electronic database search initially identified 71 articles: 21 in PubMed, 2 in Embase, 2 in Cochrane, 39 in Scopus, and 9 in Web of Science. Thirty-three duplicate records were discarded. After screening the titles and abstracts, 18 articles were excluded for not meeting the eligibility criteria. The manual reference search of the included studies did not yield any additional records. 20 articles were evaluated in full text, and all of them were ultimately included in the review. The PRISMA 2020 flow diagram representing the study selection process is illustrated in Figure 1.

### 3.2. Study Methodology

Ten articles studied etidronic acid in continuous chelation [39,40,41,42,43,44,45,46,47,48] (Table 1), and eleven articles studied it as a replacement for EDTA as a final irrigant [48,49,50,51,52,53,54,55,56,57,58] (Table 2). One study [48] evaluated both modalities.

Most studies used premolars as their samples [40,41,46,47,49,50,51,52,53,54,55,56,57,58]. Other samples used were: palatal roots of upper molars [43,44], upper central incisors [39,48], incisors, canines and premolars [45], and unspecified single-rooted teeth [42]. The mean sample size was 46.6 across the included studies (ranging from 20 [50] to 78 [58]) and the mean sample size per group was 11.3 (ranging from 5 [48,50] to 20 [53]) The majority of studies segmented the crowns from the teeth using a diamond disc [39,41,43,44,46,47,48,49,50,51,52,53,54,55,56,57,58]. The final lengths of the samples ranged from 12 mm [41,43,44,46] to 17 mm [49,56]. Two studies did not separate the crown from the root of their samples [40,42]. Lastly, Razumova et al. (2025) [45] did not specify this aspect of sample preparation.

Six articles employed a specific system for closing the apical foramen of the samples [39,40,42,47,53,57]. Five of them did so by placing wax in the apical third of the root [39,42,47,53,57]. Espinoza et al., (2021) also embedded the samples in a matrix of hydrophilic vinyl-polysiloxane impression material of regular consistency (Garant Imprint II, 3M ESPE, Madrid, Spain) [39]. Castagnola et al. (2024) closed the apex with PTFE tape and embedded the samples in putty-consistency siloxane impression material (DuoSil Putty Set; Bukwang, Busan, Republic of Korea) [40]. All articles that mentioned working length determination did so with a manual K file No. 10 [39,40,43,44,46,47,57] or 15 [42,51,52,54,55]. Most subtracted 1 mm from the length at which the file was visible at the apex [39,41,43,44,46,47,51,52,54,55,57]. Two articles did not subtract any millimeters from this length [40,42]. Eight articles did not mention the method used to determine the working length [41,45,48,49,50,53,56,58].

A total of eight different rotary systems were used in the articles included: Protaper [48,50,51,52,55,57] (Dentsply Sirona Endodontics, Tulsa, OK, USA), Protaper Next [42,43,44,58] (Dentsly-Maillefer, Ballaigues, Switzerland), Mtwo [40,53] (VDW, Munich, Germany), Protaper Gold [39] (Dentsly-Maillefer, Ballaigues, Switzerland), Profile [41] (Dentsply Maillefer, Switzerland), Wave One Gold [47] (Dentsply-Maillefer, Ballaigues, Switzerland), XPS [54] (FKG Dentaire SA, La Chaux-de-Fonds, Switzerland) and S-flexi [45] (Geosoft Endoline, Moscow, Russia). Two studies performed manual instrumentation of the root canals [49,56] and one study did not mention the instrumentation system used [46]. The most commonly used final apical caliber was 30 [42,43,44,50,51,54,55,57], ranging from 25 [47] to 50 [52].

The ten articles that tested etidronic acid in continuous chelation used it at a concentration of 9% [39,40,41,42,43,44,45,46,47,48], and one study also evaluated 18% [46]. Six studies [40,42,43,44,47,48] used the commercial presentation of etidronic acid HEDP Dual Rinse (Medcem, Weinfelden, Switzerland). Another study [46] used the commercial product Chloroquick (Innovationsendo, Nashik, India). NaOCl in combination with etidronic acid was used at concentrations of 6% [40], 5.25% [46], 3% [39,42,43,44,45,47], 2.5% [48] and 1% [41]. All studies used 17% EDTA.

In studies that used etidronic acid as the final irrigant, the samples were treated with NaOCl at a concentration of 5.25% [40,51,53,54], 2.5% [48,55,56,57,58] and 1% [52]. Etidronic acid was used as a final irrigant at a concentration of 18% in six articles [49,50,51,53,55,56], 9% in two articles [48,54] and both concentrations in three articles [52,57,58]. Another study [51] used the commercial product Chloroquick (Innovationsendo, Nashik, India). Lastly, another study [54] used TWEN KLEEN (Maarc dental, Maharashtra, India). All used 17% EDTA, except Mankeliya et al. (2021), who used 10% EDTA [49].

All articles that mentioned the irrigation method used a manual syringe [39,40,42,43,44,46,47,48,49,51,52,53,54,55,56,57,58]. Espinoza et al. (2021) connected the manual syringe to the NE-300 ‘Just infusion’ infusion system (New Era Pump Systems Inc., Farmingdale, NY, USA), which maintained a constant flow of 3 mL/min [39]. Three studies [41,45,50] did not mention the irrigation method used. Most articles used a 30-gauge needle attached to a manual syringe [40,42,43,44,46,51,52,53,54,55,57,58]. Three studies used a 27-gauge needle [39,47,48]. Two articles did not mention the needle gauge used [49,56]. Most used it by subtracting 1 or 2 mm from the working length [39,40,41,42,43,44,46,48,51,52,54,55,57]. Three studies introduced the manual syringe to its full working length [49,56,58].

The most commonly used irrigant volume during instrumentation was 2 mL after between files [40,42,43,44,45,46,47,51,53,54,55,58]. Other volumes used were 4 mL [48], 3 mL [57] and 1.5 mL [39]. 4 studies did not specify irrigant volume [41,49,50,52]. The time spent on irrigation after each file was 30 s [39,40,42,45,47], 1 min [43,44] or 2 min [48]. 12 studies did not specify irrigation time [41,46,49,50,51,52,53,54,55,56,57,58]. The most common final irrigation volume was 5 mL of irrigant [41,43,44,51,52,53,54,55,56,58]. Other volumes used were 6 mL [40], 4 mL [48], 3 mL [46] and 2.5 mL [57]. 4 studies did not specify final irrigation volume [39,45,49,50]. The time taken for the final irrigation was 5 min [52], 3 min [41,47,51,58], 2 min [40,48,53] and 1 min [43,44,56,57]. 12 studies did not specify final irrigation time [39,42,45,46,49,50,54].

4 studies [39,43,45,47] used a method of activating the irrigant, all within the continuous chelation group. The activation methods studied were: passive ultrasonic irrigation [39,43], EndoActivator [43] (Dentsply Maillefer, Ballaigues, Switzerland), EQ-S [45] (Meta Biomed, Chungcheolngnam-do, Republic of Korea), XP Endo-Finisher file [39] (FKG, La Chaux-de-Fonds, Switzerland), laser [43] and EDDY [47] (VDW, Munich, Germany).

17 articles took photographs of the three root sections under a scanning electron microscope [39,40,41,42,43,44,45,46,47,48,50,52,54,55,56,57,58]. A study evaluated the middle and apical thirds [53] and two studies evaluated only the apical third [49,51]. The most common magnification at which images were evaluated under the scanning electron microscope was 2000×, used by 8 studies [46,49,50,52,53,56,57,58]. The other magnifications used were 4000 [55], 2500 [43,48], 1500 [44], 1000 [40,41,42,47,54], 500 [51], and 100 and 800× [39]. One study [45] did not specify the magnification.

The most commonly used scale to evaluate the results was that of Hülsmann et al. (1997) [59], used by 9 studies [42,43,44,45,50,51,54,55,57], followed by that of Torabinejad et al. (2003) [60], used by 5 studies [46,49,53,56,58]. The following scales were also used: Lee et al. (2004) [61] used by Espinoza et al., 2021 [39], Gutman et al. (1994) [62] used by Castagnola et al., 2024 [40], Habshi et al. (2023) [63] used by Hazar & Hazar (2025) [48], Spano et al., 2009 [64] used by Yadav et al., (2017) [52] and an adaptation of Gambarini y Laszkiewicz (2002) [65] and Kato et al. (2016) [66] used by Aoun et al., 2023 [47]. In all of them, the lower the score, the lower the quantity of smear layer. Lottanti et al., (2009) [41] did not use scoring scales, but rather expressed the results as a percentage of the image covered with smear layer in relation to the total image.

Eight articles studied additional variables besides the removal of the smear layer. The variables studied were: the effect of the irrigant on the microhardness of dentine [43,48], erosion of root dentin [41,42], the irrigants’ antibacterial action against *Candida albicans* and *Enterococcus faecalis* [40], sealer penetration [53], push out bond strength [44,48] and marginal adaptation [57].

### 3.3. Quality Assessment Results

The results of the quality assessment of the included studies are presented in Table 3.

The average item compliance of the included studies was 58%. The maximum score was 73% (obtained by two studies [44,47]) and the minimum was 47% (obtained by two studies [45,57]). All studies provided an appropriate context and justification for the study, defined objectives and/or hypotheses, and adequately described the variables studied and the statistical calculation used. Six articles described the sample size calculation [39,43,44,47,48,50]. Eight articles addressed limitations and potential sources of bias [43,44,47,48,49,50,52,56]. No study described the method used to randomise the sample or provided access to the full study protocol.

### 3.4. Study Results

From the 10 articles that used etidronic acid as continuous chelation, 4 obtained significantly better results in terms of smear layer removal with its use than with traditional sequential irrigation [45,46,47,48]. 5 studies found no significant differences between the two options [39,41,42,43,44]. 1 study obtained better results with traditional sequential irrigation, with a statistically significant difference in the middle third of the root [40].

From the 11 articles that used etidronic acid as a final irrigant, 6 obtained significantly better results in terms of smear layer removal than traditional sequential irrigation [48,51,52,53,54,55]. From these, the difference was significant for two studies in the coronal third [48,55], 2 in the middle third [52,53] and 5 in the apical third [51,52,53,54,55]. A study found no significant differences between the two options [49]. In contrast, three studies obtained significantly better results with the use of etidronic acid as a final irrigant [50,57,58].

Regarding the studies which evaluated an additional variable, continuous chelation obtained statistically significant (*p* < 0.05) better results in all variables studied: less effect of the irrigant on dentine microhardness [43,48], lower root dentin erosion [41], greater antibacterial action against *Candida albicans* and *Enterococcus faecalis* [40] and greater push out bond strength [44,48], except in one study [42] where there were no statistically significant differences in the dentinal erosion produced. Regarding the use of etidronic acid as a final irrigant, one study obtained better marginal adaptation of the endodontic sealer than with the use of sequential irrigation [57] (*p* < 0.05). Another study found no differences in dentin microhardness or push out bond strength [48] and another observed a lower penetration of the endodontic sealer [53] (*p* < 0.05).

## 4. Discussion

The ideal endodontic irrigant should be capable of removing the smear layer from the intracanal walls and have a high antibacterial effect, without damaging the root dentin [67]. The aim of this systematic review was to compare the ability of traditional sequential irrigation (NaOCl and EDTA) to remove dentinal debris with that of etidronic acid irrigation (combined with NaOCl in continuous chelation or as a final irrigant).

The results from the included studies indicate that etidronic acid used in continuous chelation is as effective as or more effective than irrigation with NaOCl and EDTA. Thus, combining etidronic acid with NaOCl not only does not affect the properties of the latter [68] but may even enhance them [69].

Furthermore, in the literature, continuous chelation with etidronic acid has surpassed sequential irrigation in various variables, such as a reduced erosive effect on root dentin [70] or improved endodontic sealer penetration [71,72,73], so it could be a potential alternative to the traditional sequential irrigation sequence.

Two recent publications reported results similar to those obtained in the present systematic review. The first one was a systematic review [74] that analyzed several chelating agents (EDTA, HEDP, citric acid, peracetic acid and maleic acid) in terms of their efficacy, erosive potential, cytotoxicity, interaction, antimicrobial effect, impact on sealers adhesion, and release of growth factors. It concluded that etidronic acid used as continuous chelation was a promising option although further evidence is required. The second publication was a scoping review [75] that evaluated smear layer and hard-tissue debris removal, antimicrobial efficacy, and dentine erosion induced by continuous chelation. It concluded that continuous chelation was equally or more effective across all evaluated outcomes when compared with the traditional sequential protocol. To the author’s knowledge, this is the first systematic review to evaluate the efficacy of etidronic acid in smear layer removal, when used both as continuous chelation and as a final irrigant.

The results of this systematic review indicate that replacing EDTA with etidronic acid as a final irrigant results in reduced dentinal debris removal. This can be explained by the fact that etidronic acid is a weaker chelating agent than EDTA. The difference was particularly significant in the apical third. The literature reports that chelating agents are less effective in the apical third [76,77]. This is possibly due to less contact time with the root dentine and a more complex root anatomy [78], a higher proportion of sclerotic dentin [79] or a smaller canal diameter, which restricts irrigant flow [80].

The method used to study dentinal tubules in the studies included in this systematic review was the sectioning of the roots and observation under a scanning electron microscope. Subsequently, all articles except one [41] employed scales to score the amount of smear layer present. There are doubts in the literature about the validity of this technique for evaluating the efficacy of irrigants [81]. The main criticisms focus on its destructive nature, which makes it impossible to compare the sample before and after the irrigant has been applied [82], the areas of root dentin observed may not be representative of the entire sample [83], and that neither the magnification used to evaluate the images nor the scoring scales employed are standardised, and these are inherently subjective [84]. In the literature, other methods have been proposed for studying the smear layer, such as computerized microtomography [85] or in situ optical microscopy [86]. However, scanning electron microscopy is the most widely used method and was therefore considered the most appropriate for comparing results in this systematic review.

Although this systematic review is based on in vitro studies, the results may have relevant implications for the clinical performance of endodontic treatments. Smear layer removal has been associated with improved penetration of irrigants and intracanal medicaments [6,7], enhanced antimicrobial effectiveness [8], and better adaptation and sealing ability of root canal filling materials [9], all of which may influence treatment success [10]. In this context, continuous chelation with etidronic acid may represent a clinically relevant alternative to traditional sequential irrigation, as it provides comparable or superior smear layer removal while simplifying the irrigation protocol. Simplified protocols may facilitate more consistent clinical execution and reduce the risk of deviations during irrigation procedures, which has been highlighted as a relevant aspect in contemporary endodontic practice [67]. Nevertheless, these potential clinical implications should be interpreted with caution, as in vitro models cannot fully replicate the biological, anatomical, and host-related factors present in vivo. Therefore, although smear layer removal is widely regarded as a surrogate outcome related to treatment performance, well-designed clinical studies are required to determine whether the differences observed tween irrigation strategies translate into improved clinical outcomes.

It is worth mentioning the heterogeneity in certain aspects of the methodology used by the articles. They differ in aspects that have proven to be relevant to the study of the smear layer, such as whether or not to close the apex of the teeth during irrigation [87,88], the file system used [89,90] or the duration of the irrigation [91,92]. These discrepancies hinder the quantitative analysis of the data through a meta-analysis. However, there are also similarities in the methodology used. All studies used 9% etidronic acid in continuous chelation and compared it with 17% EDTA [39,40,41,42,43,44,45,46,47,48]. Also, the irrigation method used by most articles was a manual syringe and a 30 G needle at 1 or 2 mm of working length [40,42,43,44,46,51,52,54,55,57], and most articles used the same volume of irrigant during final irrigation (5 mL) [51,52,53,54,55,56,58].

Regarding the quality of the evidence, the average compliance of the studies with the quality tool was 58%. The main shortcomings were failure to calculate the sample size, failure to randomise the sample, failure to mention the limitations of the study, and failure to provide access to the full study protocol.

Lastly, with regard to the possible limitations of the present study, it should be highlighted that the search strategy and study selection were devised to include all studies that could provide data to answer the research question, but no grey literature nor conference proceedings were assessed to ensure the replicability of our methodology. Thus, additional data may be available from non-assessed sources. The results of this systematic review should be interpreted with caution. On the one hand, the in vitro nature of the studies prevents the results from being directly extrapolated to clinical practice. On the other hand, the heterogeneity in the methodology mentioned above (variations in concentration, contact time, activation methods, etc.), as well as the potential sources of bias detected, limit the quality of the evidence. Further studies employing standardized protocols are needed to confirm or refute the findings of this systematic review.

## 5. Conclusions

Based on the currently available in vitro evidence, etidronic acid used as a final irrigant removes less smear layer than EDTA, especially in the apical third. However, etidronic acid used in continuous chelation is as effective as, or more effective than, traditional sequential irrigation.

## Figures and Tables

**Table 1:** Characteristics of the included studies comparing sequential irrigation (sodium hypochlorite during instrumentation and EDTA as the final irrigant) versus continuous chelation (sodium hypochlorite in combination with etidronic acid throughout the process).

Author, Year	Sample Characteristics			Sample Preparation		Instrumentation			Irrigants	Irrigation				Results Analysis	
	Total Size	Size per Group	Teeth	Decoronation	Apical Foramen Closure	Working Length Determination	File System	Final Apical Caliber		Method	Volume and Time (Instrumentation)	Volume and Time (Final)	Activation	Measurement Scale	Magnification
Espinoza et al., 2001 [39]	52	12	Upper CI	16 mm	Yes	K-file n°10 WL–1 mm	PG	40	3%NaOCl/17%EDTA 3%NaOCl + 9%HEDP	NE-300 Syringe “Just Infusion” 3 mL/min 27 G WL–2 mm	1.5 mL 30 s	12 mL NS	XP or PUI	Lee (0–3)	100 and 800
Castagnola et al., 2024 [40]	30	10	Upper and lower PM	No	Yes	K-file n°10 to WL	Mtwo	40	6%NaOCl/17%EDTA 6%NaOCl + 9%HEDP DR 6%NaOCl/Triton	MS 30 G WL–1 mm	2 mL 30 s	6 mL 2 min	No	Gutmann (1–4)	1000
Lottanti et al., 2009 [41]	48	12	PM	12 mm	NS	NS, WL–1 mm	Profile	45	1%NaOCl/17%EDTA 1%NaOCl + 9%HEDP 1%NaOCl/2.25%peracetic acid	NS WL–1 mm	10 mL 15 min	5 mL 3 min	No	-	1000
Kfir et al., 2020 [42]	40	20	SR	No	Yes	K-file n°15 to WL	PN	30	3%NaOCl/17%EDTA 3%NaOCl + 9%HEDP DR	MS 30 G WL–2 mm	2 mL 30 s	2 mL NS	No	Hülsmann (1–4)	1000
Kamil et al., 2025 [43]	64	8	Palatal root upper M	12 mm	NS	K-file n°10 WL–1 mm	PN	30	3%NaOCl/17%EDTA 3%NaOCl + 9%HEDP DR	MS 30 G WL–2 mm	2 mL 1 min	5 mL 1 min	Endoactivator, laser, PUI	Hülsmann (1–4)	2500
Adham et al., 2023 [44]	32	8	Palatal root upper M	12 mm	NS	K-file n°10 WL–1 mm	PN	30	3%NaOCl/17%EDTA 3%NaOCl + 9%HEDP DR	MS 30 G WL–2 mm	2 mL 1 min	5 mL 1 min	No	Hülsmann (1–4)	1500
Razumova et al., 2025 [45]	30	10	I, C, PM	NS	NS	NS	S-flexi	35	3%NaOCl/17%EDTA 3%NaOCl + 9%HEDP	NS	2 mL 30 s	NS	EQ-S sonic endoactivator	Hülsmann (1–4)	NS
Hedge et al., 2019 [46]	60	12	PM	12 mm	NS	K-file n°10 WL–1 mm	NS	NS	5.25%NaOCl/17%EDTA 5.25%NaOCl/Smear Clear 5.25%NaOCl/EDTA/CHX/surfact 5.25%NaOCl + HEDP 9% and 18%Ch	MS 30 G WL–1–2 mm	2 mL NS	3 mL NS	No	Torabinejad (1–3)	2000
Aoun et al., 2023 [47]	75	15	Lower PM	15 mm	Yes	K-file n°10 WL–1 mm	Wave One Gold	25	3%NaOCl/17%EDTA 3%NaOCl + 9%HEDP DR	MS 27 G WL–2 mm	2 mL 30 s	6 mL 3 min	EDDY	Adaptation of Gambarini and Laszkiewicz and Kato et al. (1–4)	1000
Hazar & Hazar, 2025 [48]	30	5	Upper CI	15 mm	No	NS	Protaper	40	2.5%NaOCl 2.5%NaOCl/17%EDTA 2.5NaOCl/9%HEDP DR 2.5%NaOCl/phytic acid 2.5%NaOCl + 9%HEDP DR 2.5%NaOCl + phytic acid	27 G WL–1 mm	4 mL 2 min	4 mL 2 min	No	Habshi (1–4)	2500

WL: working length, CI: central incisors, I: incisors; C: canines; PM: premolars; M: molars; SR: single rooted teeth; NS: not specified, PG: Protaper Gold; PN: Protaper Next; EDTA: ethylenediaminetetraacetic acid; HEDP: etidronic acid; CHX: chlorhexidine; DR: Dual Rinse; Ch: Chloroquick; MS: manual syringe; G: gauge; PUI: passive ultrasonic irrigation.

**Table 2:** Characteristics of the included studies comparing EDTA versus etidronic acid as a final irrigant.

Author, Year	Sample Characteristics			Sample Preparation		Instrumentation			Irrigants Size per Group	Irrigation				Results Analysis		
	Total Size	Size per Group	Teeth	Decoronation	Apical Foramen Closure	Working Length Determination	Instrumentation System	Total Size		Method	Volume and Time (Instrumentation)	Volume and Time (Final)	Activation	Measurement Scale	Analysis per Thirds	Magnification
Mankeliya et al., 2021 [49]	60	15	Lower PM	17 mm	NS	NS	Manual	40	5.25%NaOCl/10%EDTA 5.25%NaOCl/10%citric acid 5.25%NaOCl/18%HEDP 5.25%NaOCl/7%maleic acid	MS WL	NS	NS	No	Torabinejad (1–3)	Only apical	2000
Pendalwar et al., 2024 [50]	20	5	Lower PM	Yes, NS	No	NS	Protaper	30	NaOCl/17%EDTA NaOCl/18%HEDP NaOCl/0.2%Chitosan	NS	NS	NS	No	Hülsmann (1–4)	Yes	2000
Patil et al., 2018 [51]	40	10	Lower PM	14 mm	NS	K-file n°15 WL–1 mm	Protaper	30	5.25%NaOCl/17%EDTA 5.25%NaOCl/18%HEDP Ch high 5.25%NaOCl/Biopure MTAD	MS 30 G WL–1 mm	2 mL NS	5 mL 3 min	No	Hülsmann (1–4)	Only apical	500
Yadav et al., 2017 [52]	50	10	Lower PM	Yes, NS	NS	K-file n°10 or n°15 WL–1 mm	Protaper	50	1%NaOCl/SC 1%NaOCl/9% and 18%HEDP 1%NaOCl/Biopure MTAD	MS 30 G WL–1,2 mm	NS	5 mL 5 min	No	Spano (1–3)	Yes	2000
Kour et al., 2019 [53]	60	20	Lower PM	16 mm	Yes	NS	Mtwo	35	5.25%NaOCl/17%EDTA 5.25%NaOCl/18%HEDP 5.25%NaOCl/Biopure MTAD	MS 30 G NS	2 mL NS	5 mL 2 min	No	Torabinejad (1–3)	Apical and middle	2000
Varma et al., 2023 [54]	33	11	Lower PM	14 mm	No	K-file n°15 WL–1 mm	XPS	30	3%NaOCl/17%EDTA 3%NaOCl/9%HEDP (TK) 3%NaOCl/Remix 2in1	MS 30 G WL–1 mm	2 mL NS	5 mL NS	No	Hülsmann (1–4)	Yes	1000
Bajpe et al., 2023 [55]	30	10	Lower PM	16 mm	No	K-file n°15 WL–1 mm	Protaper	30	2.5%NaOCl/17%EDTA 2.5%NaOCl/18%HEDP 2.5%NaOCl/0.2%chitosan	MS 30 G WL–2 mm	2 mL NS	5 mL 3 min	No	Hülsmann (1–4)	Yes	4000
Kuruvilla et al., 2015 [56]	30	10	Lower PM	17 mm	No	NS	Manual	40	2.5%NaOCl/17%EDTA 2.5%NaOCl/18%HEDP 2.5%NaOCl/7%maleic acid	MS WL	NS	5 mL 1 min	No	Torabinejad (1–3)	Yes	2000
Hazar & Hazar, 2025 [48]	30	5	Upper CI	15 mm	No	NS	Protaper	40	2.5%NaOCl 2.5%NaOCl/17%EDTA 2.5%NaOCl/9%HEDP DR 2.5%NaOCl/phytic acid 2.5%NaOCl + 9%HEDP DR 2.5%NaOCl + phytic acid	MS 27 G WL–1 mm	4 mL 2 min	4 mL 2 min	No	Habshi (1–4)	Yes	2500
Ulusoy et al., 2017 [57]	70	10	Lower PM	14 mm	Yes	K-file n°10 WL–1 mm	Protaper	30	2.5%NaOCl/17%EDTA 2.5%NaOCl/9% and 18%HEDP 2.5%NaOCl/0.5%, 1% and 2%peracetic acid	MS 30 G WL–1–2 mm	3 mL NS	2,5 mL 1 min	No	Hülsmann (1–4)	Yes	2000
Erik et al., 2019 [58]	78	13	Lower PM	14 mm	No	NS	Protaper Next	40	2.5%NaOCl/17%EDTA 2.5%NaOCl/9%and 18%HEBP 1%NaOCl/9%HEBP 2%NaOCl/18%HEBP	MS 30 G WL	2 mL NS	5 mL 3 min	No	Torabinejad (1–3)	Yes	2000

WL: working length; PM: premolars; NS: not specified; EDTA: ethylenediaminetetraacetic acid; HEDP: etidronic acid; Ch: Chloroquick; TK: Tween Kleen; MS: manual syringe; G: gauge.

**Table 3:** Results of the risk of bias assessment according to the modified CONSORT scale for in vitro studies on dental materials.

	Espinoza et al., 2021 [39]	Castagnola et al., 2024 [40]	Lottanti et al., 2009 [41]	Kfir et al., 2020 [42]	Kamil et al., 2025 [43]	Adham et al., 2023 [44]	Razumova et al., 2025 [45]	Hegde et al., 2019 [46]	Aoun et al., 2023 [47]	Hazar & Hazar, 2025 [48]	Mankeliya et al., 2021 [49]	Pendalwar et al., 2014 [50]	Patil et al., 2018 [51]	Yadav et al, 2017 [52]	Kour et al., 2019 [53]	Varma et al., 2023 [54]	Bajpe et al., 2023 [55]	Kuruvilla et al., 2015 [56]	Ulusoy et al., 2017 [57]	Erik et al., 2019 [58]	Yes (%)	No (%)
1	Y	Y	Y	Y	Y	Y	Y	Y	Y	N	Y	Y	Y	Y	Y	Y	Y	N	N	N	81	19
2a	Y	Y	Y	Y	Y	Y	Y	Y	Y	Y	Y	Y	Y	Y	Y	Y	Y	Y	Y	Y	100	0
2b	Y	Y	Y	Y	Y	Y	Y	Y	Y	Y	Y	Y	Y	Y	Y	Y	Y	Y	Y	Y	100	0
3	Y	Y	Y	Y	Y	Y	N	Y	Y	Y	N	N	Y	Y	Y	Y	Y	Y	Y	Y	86	14
4	Y	Y	Y	Y	Y	Y	Y	Y	Y	Y	Y	Y	Y	Y	Y	Y	Y	Y	Y	Y	100	0
5	Y	N	N	N	Y	Y	N	N	Y	Y	N	Y	N	N	N	N	N	N	N	N	33	67
6	N	N	Y	N	N	Y	N	N	N	N	N	N	N	N	N	N	N	N	N	N	9	91
7	N	N	N	N	N	N	N	N	N	N	N	N	N	N	N	N	N	N	N	N	0	100
8	N	N	N	N	N	N	N	N	N	N	N	N	N	N	N	N	N	N	N	N	0	100
9	N	Y	N	Y	N	N	N	Y	Y	N	Y	Y	Y	Y	N	Y	Y	Y	N	Y	62	38
10	Y	Y	Y	Y	Y	Y	Y	Y	Y	Y	Y	Y	Y	Y	Y	Y	Y	Y	Y	Y	100	0
11	Y	N	Y	Y	Y	Y	Y	Y	Y	Y	Y	Y	Y	N	Y	Y	Y	N	Y	Y	81	19
12	N	N	N	N	Y	Y	N	N	Y	Y	Y	Y	N	Y	N	N	N	Y	N	N	38	62
13	Y	Y	N	Y	Y	Y	Y	Y	Y	Y	Y	N	Y	N	Y	N	Y	Y	Y	Y	76	24
14	N	N	N	N	N	N	N	N	N	N	N	N	N	N	N	N	N	N	N	N	0	100
%	60	53	53	60	67	73	47	60	73	60	60	60	60	53	53	53	60	53	47	53	-	-

Y: fulfilled; N: non-fulfilled.

## Data Availability

The original contributions presented in this study are included in the article. Further inquiries can be directed to the corresponding author.

## Supplementary Materials

The following supporting information can be downloaded at: https://www.mdpi.com/article/10.3390/jfb17010048/s1, Table S1: Search strategy (Database, search string and number of results).
