# Supplementary material for: Efficacy of Etidronic Acid for Smear Layer Removal: A Systematic Review of In Vitro Studies
Source: J Funct Biomater. 2026 Jan 16;17(1):48. doi: 10.3390/jfb17010048 (PMC12842351; doi:10.3390/jfb17010048)
Supplement: Supplementary file 1 [file jfb-17-00048-s001.zip › jfb-4082424-supplementary.pdf]

Supplementary Table S1. Search strategy and findings per database

Database	Search string	Results
Medline	#1 All fields ("root canal treatment"[MeSH Terms] OR endodontics[MeSH Terms])<br>#2 All fields ("etidronic acid"[MeSH Terms] OR HEDP OR HEBP)<br>#3 All fields ("chelation therapy" OR "chelating agents"[MeSH Terms] OR "root canal irrigants"[MeSH Terms] OR "smear layer"[MeSH Terms])<br>#4 All fields “in vitro”<br>#1 AND #2 AND #3 AND #4	46.066<br>3.243<br>42.381<br><br>1.873.085<br>**21**
Embase	#1 root canal treatment”:ab,ti OR endodontics:ab,ti<br>#2 "etidronic acid":ab,ti OR HEDP:ab,ti OR HEBP:ab,ti<br>#3 "chelation therapy":ab,ti OR "chelating agents":ab,ti OR "root canal irrigants":ab,ti OR "smear layer":ab,ti<br>#4 “in vitro”:ab,ti<br>#1 AND #2 AND #3 AND #4	7.089<br>784<br>13.481<br>1.972.874<br>**2**
Cochrane	#1 All text: "root canal treatment" OR endodontics<br>#2 All text: "etidronic acid" OR HEDP OR HEBP<br>#3 All text: "chelation therapy" OR "chelating agents" OR "root canal irrigants" OR "smear layer"<br>#4 All text: “in vitro”<br>#1 AND #2 AND #3 AND #4	5.492<br>608<br>2.009<br>26.521<br>**2**
Scopus	#1 TITLE-ABS-KEY ("root canal treatment") OR TITLE-ABS-KEY (endodontics)<br>#2 TITLE-ABS-KEY ("etidronic acid") OR TITLE-ABS-KEY (HEDP) OR TITLE-ABS-KEY (HEBP)<br>#3 TITLE-ABS-KEY ("chelation therapy") OR TITLE-ABS-KEY ("chelating agents") OR TITLE-ABS-KEY ("root canal irrigants") OR TITLE-ABS-KEY ("smear layer")<br>#4 TITLE-ABS-KEY (“in vitro”)<br>#1 AND #2 AND #3 AND #4	181.714<br>11.384<br>64.488<br><br>2.772.330<br>**39**
Web of Science	#1 Topic ("root canal treatment" OR "endodontics")<br>#2 Topic ("etidronic acid" OR "HEDP" OR "HEBP")<br>#3 Topic "chelation therapy" OR "chelating agents" OR "root canal irrigants" OR "smear layer")<br>#4 Topic (“in vitro”)<br>#1 AND #2 AND #3 AND #4	27.626<br>4.500<br>62.585<br>3.535.441<br>**9**
